# Stories From the Field: The Use of Information and Communication Technologies to Address the Health Needs of Underserved Populations in Latin America and the Caribbean

**DOI:** 10.2196/publichealth.4108

**Published:** 2015-03-17

**Authors:** Nasim Farach, Gladys Faba, Soroya Julian, Felipe Mejía, Báltica Cabieses, Marcelo D'Agostino, Andrea A Cortinois

**Affiliations:** ^1^ Public eHealth, Innovation & Equity in Latin America & the Caribbean (eSAC) project Tegucigalpa Honduras; ^2^ Public eHealth, Innovation & Equity in Latin America & the Caribbean (eSAC) project Tlayacapan Mexico; ^3^ Public eHealth, Innovation & Equity in Latin America & the Caribbean (eSAC) project Kingston Jamaica; ^4^ Public eHealth, Innovation & Equity in Latin America & the Caribbean (eSAC) project Bogotá Colombia; ^5^ Clínica Alemana Faculty of Medicine Universidad del Desarrollo Santiago Chile; ^6^ Pan American Health Organization (PAHO) Washington, DC United States; ^7^ Dalla Lana School of Public Health University of Toronto Toronto, ON Canada

**Keywords:** eHealth, Latin America, vulnerable populations, qualitative research

## Abstract

**Background:**

As their availability grew exponentially in the last 20 years, the use of information and communication technologies (ICT) in health has been widely espoused, with many emphasizing their potential to decrease health inequities. Nonetheless, there is scarce availability of information regarding ICT as tools to further equity in health, specifically in Latin American and Caribbean settings.

**Objective:**

Our aim was to identify initiatives that used ICT to address the health needs of underserved populations in Latin America and Caribbean. Among these projects, explore the rationale behind the selection of ICT as a key component, probe perceptions regarding contributions to health equity, and describe the challenges faced during implementation.

**Methods:**

We conducted an exploratory qualitative study. Interviews were completed via Skype or face-to-face meetings using a semistructured interview guide. Following participant consent, interviews were audio recorded and verbatim transcriptions were developed. All transcriptions were coded using ATLASti7 software. The text was analyzed for patterns, shared themes, and diverging opinions. Emerging findings were reviewed by all interviewers and shared with participants for feedback.

**Results:**

We interviewed representatives from eight organizations in six Latin American and Caribbean countries that prominently employed ICT in health communication, advocacy, or surveillance projects. ICT expanded project's geographic coverage, increased their reach into marginalized or hard-to-reach groups, and allowed real-time data collection. Perceptions of contributions to health equity resided mainly in the provision of health information and linkage to health services to members of groups experiencing greater morbidity because of poverty, remote place of residence, lack of relevant public programs, and/or stigma and discrimination, and in more timely responses by authorities to the health needs of these groups as a result of the increased availability of strategic information on morbidity and its social determinants. Most projects faced initial resistance to implementation because of lack of precedents. Their financial and technical sustainability was threatened by reliance on external funding and weak transitional structures amidst key staff changes. Projects often experienced challenges in establishing meaningful communication with target audience members, mainly because of divergent motivations behind ICT use between projects and its target audience and the lack of access or familiarity with ICT among the most underserved members of such audiences.

**Conclusions:**

ICT can benefit projects focusing on the health needs of underserved populations by expanding the breadth and depth of target audience coverage and improving data management. Most projects tended to be small, short-term pilot interventions with limited engagement with the formal health sector and did not include health equity as an explicit component. Collaborative projects with government institutions, particularly those with health surveillance objectives, seemed to be the most optimistic about long-term sustainability.

## Introduction

It is now widely accepted that social, economic, and environmental conditions are closely linked to health outcomes and that differences in these circumstances among a country’s population contribute to persistent and pervasive health inequities [1,2]. This rings particularly true to Latin America and the Caribbean (LAC), regarded as the most persistently unequal region in the world [3]. While certain reductions have been recently noted, LAC countries feature consistently among the most unequal nations [4]. Stark levels of inequality are present in various aspects of everyday life for LAC citizens, including income, housing, education, employment, and health. Differences in health outcomes exist both within and among LAC countries. A regional study in 2010 by the Latin American Center for Rural Development showed that all nations in the region have sub-national territories and lag behind the rest of the country in terms of development indicators and that they tend to have smaller populations, are more rural, and have a larger percentage of indigenous or Afro-descendent inhabitants [5].

Health inequities are closely related to varying levels of power and access to different resources, including information and communication technologies (ICT). As their availability grew exponentially in the last 20 years, the use of ICT in health, often referred to as eHealth, has been widely espoused, with many emphasizing their potential to decrease health inequities [6,7]. The 58th World Health Assembly in May 2005 adopted Resolution WHA58.28, urging member states to develop eHealth strategies and recommending the development of a strategic plan for eHealth; reaching communities, including vulnerable groups; and evaluating and sharing knowledge about eHealth activities to promote equity and equality [8].

Telehealth, in particular, has been at the forefront of the eHealth field. Defined by the World Health Organization (WHO) as the use of ICT by health care professionals for the exchange of information for diagnosis, treatment and prevention of disease and injuries, research and evaluation, and continuing education, telehealth has been one of the most visible, documented, and celebrated applications of ICT in health. Despite this, leading experts in health inequities emphasize the critical importance of addressing social determinants of health (social determinants of health) beyond exclusive clinical settings. For instance, the Strategic Review of Health Inequities in England, chaired by Michael Marmot, unequivocally endorsed the need to address social determinants from a broader perspective to effectively reduce health inequities [9]. Similarly, the Final Report of the WHO Commission on social determinants of health proposed three overarching recommendations that equally highlighted the critical importance of non-clinical factors in health outcomes: improve the conditions of daily life; tackle the inequitable distribution of power, money, and resources; and expand the knowledge base, develop a workforce that is trained in social determinants of health, and raise public awareness about social determinants of health [10].

These calls for action emphasize a need to move beyond purely clinical activities in addressing health inequity by establishing a clear link between social determinants of health and public health activities. As expressed by WHO Director General, Margaret Chan, “in its traditional concern with prevention, public health has much to gain when biomedical approaches to health and disease are extended by a focus on the true root causes of ill-health, suffering and premature death” [11]. ICT may thus have a significant role to play in this extension, beyond a purely telemedical approach, by serving as a channel to effectively address social determinants of health and reduce avoidable inequities in health. This equity-focused public eHealth approach has been described in the literature. Friede, Blum, and McDonald advocated for a greater integration of ICT with public health to enhance disease prevention and health promotion in underserved populations through applications that improve surveillance systems, communication with the public, and service provision [12]. At a political level, the Ministers of Health from LAC highlighted the importance of ICT tools as they incorporated an area of action called Harnessing Knowledge, Science, and Technology for Public Health within the Health Agenda for the Americas in 2007.

With the advent of the Internet and mobile phones, a mounting body of work in the eHealth field has documented reasons for adoption as well as challenges faced by Web-based and mHealth initiatives in diverse settings [13-15]. A large part of this literature focused on clinical applications of eHealth, and recent years have seen an increase in works documenting experiences in developing nations, particularly in Africa—a departure from the initial focus in the developed world [16-19].

Nonetheless, there is currently scarce availability of information regarding ICT as tools to further equity in health specifically in LAC settings. In 2010, the Latin American Economic Commission (CEPAL) highlighted the experiences of six projects in the region that specifically dealt with ICT and inequities, presenting initiatives in six distinct clinical areas: clinical/management systems, unique personal identifiers, remote medical appointments, electronic clinical history, electronic medical prescriptions, and telemedicine. However, the report did not include examples of the use of ICT beyond clinical settings and the impact they could have on social determinants of health [20].

Furthermore, and despite widespread enthusiasm for at least the clinical applications of eHealth in diverse circles, a consensus statement disseminated by thematic experts at the WHO Global eHealth Evaluation Meeting in 2011 urged the continued identification of the barriers to undertaking and using evaluation in eHealth, make recommendations to overcome them, and identify gaps in knowledge where better evidence could increase the appropriate use, scale, and impact of eHealth in resource-limited settings [21].

In response to this knowledge gap, and as part of the Public eHealth, Innovation & Equity in Latin America and the Caribbean (eSAC) project, we conducted an exploratory study among eHealth initiatives in LAC to assess the rationale behind the selection of ICT as a significant project component, probe perceptions regarding contributions to health equity, and describe the challenges these projects faced when conducting their activities. This paper presents the main results of this study in an attempt to capture the experience of projects whose activities dealt with what we considered a particularly understudied purpose (focused on health of vulnerable groups) in a particularly understudied setting (Latin America and the Caribbean). The eSAC project, a joint initiative between the University of Toronto and the Pan American Health Organization (PAHO) and funded by the International Development Research Centre (IDRC), aimed to contribute to the advancement of equity in health in LAC by exploring the intersection of ICT, public health and equity, and fostering the establishment of a virtual community of practice around this intersection.

## Methods

### Sample Selection

A qualitative research design was selected to allow for an in-depth understanding of the experiences of initiatives that used ICT to address the health needs of underserved populations in LAC. A purposeful sample of projects was selected using specific criteria. To be considered, projects needed to address a public health issue in LAC, have ICT as a key element in project implementation, include an equity component, and be in implementation at the time of the study or completed during the 2 years previous to the interview. For purposes of this study, traditional media, such as radio, television, and landline telephones were not included in our definition of ICT. Because many public health programs did not explicitly mention equity in their objectives or mission statement, we considered the equity criterion to be met if the project addressed a public health need in a traditionally underserved population, such as highly stigmatized groups, rural communities, underprivileged urban areas, youth, or ethnic minorities. Programs that completed projects more than 2 years prior to the time of the interview, but that continued implementing other ICT public health interventions in the region were still considered for inclusion, given the implementing organization´s continuous engagement with public eHealth. In light of the study team’s language skills, only participants fluent in English or Spanish were considered.

### Data Collection

Potential participants were identified by a review of the mapping of eHealth projects in LAC conducted by the Public eHealth Equity and Innovation in Latin America and the Caribbean (eSAC) project, publically available at the project’s platform, as well as a scoping review of such interventions conducted in 2012/2013 as part of the eSAC project. This was completed by an expert elicitation process, a structured approach to systematically consult experts on uncertain issues [22]. We contacted four subject-matter experts in the eHealth and equity field in LAC—three with academic backgrounds and one from an international public health agency. We shared study objectives, selection criteria and the preliminary list of participants, and asked for additional potential candidates. As a result of these activities, a list of 18 projects was developed, all of which were contacted via email message or contact request forms at project websites. Out of these, 11 projects responded and 8 agreed to participate in the study.

A semistructured interview guide was developed and piloted with representatives from two projects in Chile and Mexico. Recommendations from the pilot were incorporated into the questionnaire. Interviews were conducted by trained members of the study team using Skype or face-to-face meetings (when the interviewer and participant lived in the same city) and lasted between 45 minutes and 1 hour and 30 minutes. Interviewers followed this guide to address specific topics, including project background, objectives, activities, monitoring and evaluation practices, rationale for ICT tool selection, perception of project’s impact on health inequities, main facilitators, and challenges, and awareness of other eHealth projects. Following participant consent, interviews were audio recorded and verbatim transcriptions were developed.

### Analysis

As the interviews were transcribed, a preliminary list of primary themes was identified. These themes became the basis for the first codebook that defined each thematic code. Given transcriptions were developed in participant’s native languages (both Spanish and English), a bilingual study team member was responsible for coding the interviews. All transcriptions were inputted and coded using ATLASti7 software (Scientific Software Development Gmb). During coding, emerging codes were identified and added to the codebook. Memos were written during the process to record our impressions and reflections. Repeated reading of the transcripts facilitated familiarization with the data. Once coding was completed, text was retrieved using ATLASti query functions and analyzed for patterns, shared themes, and diverging opinions. Emerging findings were reviewed by all interviewers and shared with all participants for feedback. While three of them responded thanking the study team for their participation, no technical feedback was received.

## Results

### Sample Description

A total of 8 participants representing an equal number of projects agreed to participate. Four projects were based in South America (Colombia, n=2, and one each for Chile and Peru), two in the Caribbean (one each from Barbados, Haiti), and two from Central and North America (one each from Mexico, Guatemala). Projects predominantly addressed communicable diseases (n=5): two dealt with HIV/AIDS and one each with water-borne infections (mostly cholera), tuberculosis, and dengue fever. Two projects focused on non-communicable diseases and one dealt with juvenile bullying. While project objectives were more frequently related with health communications (n=5), such as behavior change or health promotion, two projects dealt with public health surveillance and one with advocacy and political incidence. Projects predominantly used mobile phone text messages as their main implementation channel (n=5), but two projects were mainly delivered through social media, particularly Facebook, and one through online games. International agencies constituted the main funding source for most projects (n=5), although one each were primarily supported by a local government, a private business organization or an academic organization. One project was a mixed initiative between an international agency and a private business organization. Table 1 summarizes the characteristics of participating projects, by country, fields of work, ICT channel, and funding source.

**Table 1:** Profile of participating projects, by country, fields of work, and funding source (N=8).

Characteristics			N
**By country**			
		Barbados	1
		Chile	1
		Colombia	2
		Guatemala	1
		Haiti	1
		Mexico	1
		Peru	1
**By morbidity cause**			
	**Communicable diseases**		
		Dengue fever	1
		Water-borne infection (mainly cholera)	1
		HIV/AIDS	2
		Tuberculosis	1
	**Non-communicable diseases (NCD)**		
		General NCD	1
		Smoking-related NCD	1
	**Injury**		
		Juvenile bullying	1
**By technical field of work**			
		Advocacy and political incidence	1
		Health communications	5
		Public health surveillance	2
**By main ICT channel used**			
		Mobile phone text messages	5
		Gaming (online)	1
		Social Media (mainly Facebook)	2
**By main funding source**			
		Academic	1
		International agency	5
		Mixed (Private/International agency)	1
		National government	1
**By origin of main funding source**			
		International	5
		Mixed (National and international)	1
		National	2

Results are presented around three main themes that convey key aspects of project design and implementation. First, we explore the rationale behind the selection of ICT as the main channel to implement a public health project targeting underserved populations, instead of alternative, non-ICT channels. Second, and in light of projects’ focus on underserved populations, we probe perceptions of contribution to health equity. Finally, we analyze the challenges faced by participants during project implementation and discuss the main coping strategies adopted to address these. Table 2 presents the main subthemes identified in each one of these three main themes.

**Table 2:** Main themes and subthemes: Rationale behind ICT use, perceptions of impact on health equity, and challenges to program implementation.

Main theme	Subthemes	
**Rationale behind the use of ICT for public health projects targeting underserved populations**		
	Expansion of geographic and social reach	
	Real time data management	
	Interaction enabler	
**Perceptions of impact on health equity**		
	Access to health information and services	
	Data for decision making	
	Virtual peer support	
**Challenges**		
	**Internal**	
		Lack of precedents
		Technical and financial sustainability
	**External**	
		Lack of meaningful interaction
		Unfamiliarity with ICT
		Data ownership

### Rationale Behind the Use of Information and Communication Technologies for Public Health Projects

#### Expansion of Geographic and Social Reach

The ability to expand geographic or target-audience coverage rates, often with little or no economic investment, featured prominently as a reason to select ICT as a central project component.

These powerhouses of platforms [Twitter and Facebook]…are a very effective and cheap form of communication for us because the campaign was spread over many territories. When you look at trying to advertise and communicate with such a wide audience for a small civil society organization that would be very difficult [otherwise].

ICT also increased the depth of program scope by facilitating connection with stigmatized, hard-to-reach populations—often the most vulnerable, isolated, and who suffer most from health inequities. The selection of a specific ICT varied depending on the characteristics of target audiences. Social media, Facebook in particular, were often selected when the program intended to reach younger audiences, broad national or regional constituencies, or harder-to-reach groups that shared a stigmatized behavior.

Overall coverage was minimal in groups like men who have sex with men... you will not find many of them in public venues because of stigma and discrimination. Because they don´t wish to be perceived as gay, they become hermetic, hidden. Social media have helped us most to reach this group.

The greater availability of mobile phones among economically disadvantaged and rural populations drew many projects to rely on text messages, instead of social media or mobile phone apps. In these cases, target audiences had no or limited access to computers, mobile phones, and/or Internet access.

People with tuberculosis are the most vulnerable, the poorest; we assumed that we would be able to reach them through text messages. We could have developed an app, but it would reach the youngest only and would be more complex [for users]. Our idea was to go down to basics, the most commonplace at the time, which I believe is still the text message.

Some projects adopted a mixed approach. For instance, a project used social media to communicate with members in the capital and other large urban areas, while interventions in rural and other remote regions relied predominantly on text messages.

#### Real Time Data Management

For health surveillance projects, ICT allowed for more effective survey recruitment and real time data management. This improved data accessibility, facilitated data quality assessments, and allowed for improved monitoring of program activities.

SMS text messages were the most efficient and economical way we found for data to flow from the field. The system gives access to first-hand information, directly from the field, from any corner of the country in an almost real time. Public health benefits from access to this data.

The real time ability to report and track [text messages] was one of the critical aspects of the project that was able to benefit us and if the project was to be replicated it would be something that we would definitely recommend.

#### Interaction Enabler

ICT also brought added value by allowing audience members to become active participants in content creation or communication, beyond mere recipients of information. The dynamic, self-generating characteristic of ICT was highlighted as one of its most valuable and promising features:

ICT enables users to produce content. It then becomes an [educational] material that does not end. Our idea is to get to a point where audiences can continue to create material, being a direct player in this construction.

Participants perceived that eHealth was still in its early development and that projects such as theirs represented the tip of the iceberg in regards to the field´s potential to address other public health concerns. Several participants, while sharing the enthusiasm, stressed the importance of keeping the focus on the project’s public health objectives, rather than in the ICT themselves, and stressed the need for further research and evaluation in the field.

Many times we think that the project is the technology itself, the act of sending text messages. To be honest, we could have done this [implement the project] in a different way. The good thing is that, since the beginning, we thought about the problem we faced, adherence to treatment, and that this [text messages] was the way in which we could address it in a cheaper, simpler, and more innovative way.

### Perceptions of Impact on Health Equity

#### Overview

For purposes of this study, we avoided a prescribed definition of equity. Instead, we adopted a more exploratory approach that allowed us to understand the perception of participants regarding their project’s contribution to health equity.

#### Access to Health Information and Services

Perceptions of contributions to health equity resided mainly in the provision of health information and linkage to health services to members of groups experiencing greater morbidity because of poverty, remote place of residence, lack of relevant public programs, and/or stigma and discrimination. In some cases, projects also included a component to effectively link individuals with health services. This was also considered to contribute to health equity, especially considering that many of these individuals faced barriers that had previously prevented their access to health services:

Teenagers are not included in any regular program and have no clear alternatives to quit smoking. We are reducing inequity by working with groups with little access to health, but who have a cellular phone.

#### Data for Decision Making

Among surveillance projects, participants mentioned that their activities increased the availability of strategic information on morbidity and its social determinants among underserved populations, leading to improved prevention activities and timely responses by Ministries of Health. Perception among participants was that information generated by projects served as a catalyzer for long-overdue health service provision to isolated and marginalized groups:

Now, people living in isolated and rural mountain areas are covered by the same system as people in the capital…Before, two days walks were needed to reach some communities, but now the information gets to the central post and the system generates an immediate alert.

#### Virtual Peer Support

Another perception of contribution to health equity was the development of virtual self-help groups among people with a specific ailment that eased the psychological burden of disease, created a sense of community, and contributed to improved health outcomes. This was particularly stressed by projects addressing highly stigmatized diseases, like human immunodeficiency virus (HIV):

This tool [text-based messages] provided the…confidentiality that many participants sought…Adequate emotional support [through the mHealth group] became a crucial factor in facing daily life.

Nonetheless, some participants expressed caution in the assessment of contributions to the reduction of health inequities because of the lack of robust evaluations and concerns regarding the exclusion of the most vulnerable:

The other obvious bias is that subscribers to our [text message] system are likely more familiar with technology and will perceive it as a more useful tool. This makes us ask ourselves: will we generate a greater gap in relation to the destitute, poorest, or older citizens?

### Challenges to Project Implementation

#### Overview

Participant narratives revealed both internal and external challenges when implementing eHealth interventions for public health. Internal challenges, referring to obstacles occurring within the organization, were described at different stages of project implementation: resistance to roll out at early phases and uncertainty in project impact and sustainability toward their end. External challenges, on the other hand, refer to barriers arising from projects’ interaction with its target audiences, partners and other outside stakeholders, and were experienced mostly during the implementation stage.

#### Internal Challenges

##### Lack of Precedents

Many participants faced resistance or skepticism to the introduction of eHealth activities during the initial phases of implementation. In projects operating within organizations where many employees were unfamiliar with ICT, the incorporation of a new ICT element was perceived as an increase in work load that also provided for more intense, unrelenting oversight by off-site supervisors. In other cases, initial tepid responses arose from the lack of precedents:

When people don’t understand the benefit [of using ICT] they feel they have one more task to do, that there is one more eye watching over them every day. The perception is that they are now forced to report this new information, that a new inspector is watching them on a daily basis.

##### Technical and Financial Sustainability

The lack of sustainability, or project capacity to maintain activities, products and outcomes over time, constantly featured as a significant challenge. Participant narratives described two domains of sustainability challenges: financial and technical. Funding instability loomed as a permanent challenge and became a relentless concern among participants, as international agencies were the most common source of support among sampled projects. From a technical perspective, projects faced the threat of lack of continuity amidst staffing changes, particularly when the person who was more closely linked to project implementation transitioned to another position or organization:

An important lesson is that it is important to transmit and share these experiences to give more sustainability in time beyond the people [originally implementing the project].

Faced rather frequently with these sustainability challenges, projects often recurred to the establishment of partnerships with a diverse set of stakeholders as a strategy to enhance their feasibility. These included collaborations with Ministries of Health, traditional media outlets, mobile phone service providers, community leaders, universities, and existing networks of ICT enthusiasts:

I try to invite and involve the private sector, especially those willing to engage in corporate social responsibility…I also try to involve students. I act as a talent hunter among these talented youth, highlighting the opportunity they have to build a name for themselves and showcase their talent.

Participants working in collaborative projects with government institutions were the most optimistic about long-time sustainability:

This system is not expected to end anytime; it does not have a final date. It’s a tool that’s been incorporated into the organization’s management.

This feeling was even stronger in projects that operated within settings where there was a perception of broader, higher-level support for ICT as part of national policy, instead of the result of a compartmentalized, discrete collaboration between a project and a specific government entity. Such a scenario was described by participants from Colombia:

The ICT Ministry and Ministry of Culture are very interested in promoting the use of ICT in different subjects…the stage is set for those interested in working in this, [ICT] when the state is interested in sponsoring.

The ICT Ministry has well defined objectives. If one approaches them with an idea, they are very open to it. This facilitated our entry into workplace sites [for the study].

Beyond the obvious objective of gaining financial sustainability, partnerships were also seen as a strategy to engage with communities, obtain their buy-in, and achieve project objectives. Identifying and partnering with key, local opinion leaders was associated with a greater access to implement project activities and a better response from local audiences.

We quickly identified…that the most effective way for the campaign to work is if people on the ground, within the community, take up the campaign. We were able to identify through civil society organizations some team leaders that had the time, the ability and networking to achieve things…Countries where we had strong champions, out there pushing it with their voice and speaking to their communities, we saw a much better response to the campaign.

One thing I’ve learned through this pilot is {to understand} the interaction among something new, like technology, and social structures, which tend not to vary. Even if internet is widely available, the community is still in place and is an important factor.

While partnerships with a myriad of actors featured prominently in the narratives, the establishment of such with other projects implementing public eHealth projects did not surface during the study, despite most participants being aware to some degree of other projects employing ICT in public health, particularly those implemented in the same city or surrounding areas. Very few participants mentioned initiatives beyond their country.

#### External Challenges

##### Lack of Meaningful Interaction

For behavior change interventions, the main external challenge was the difficulty of establishing meaningful communication with target audience members through social media channels. Social media was often perceived as a de-personalized channel poorly suited for establishing the often long-term interaction required for successful behavior change:

Behavior change is not easy to venture into, even in face-to-face interactions when you have a more human, closer relationship. Let alone through a computer where all sorts of manipulation can take place.

Further, participants described divergent purposes behind the use of social media among project workers and target audience members. While projects intended to transmit health-related content, the audience mainly used social media for entertainment reasons; projects reported that they could disseminate much information but receive little feedback back. Additionally, projects expressed difficulty transmitting health content in a quantity and format that complied with both project objectives and audience’s needs and interests:

The reasons why people use social media are completely different than ours, which are more linked to health, prevention, or education. Many use these media to meet [sexual partners], interact with friends, or establish relationships.

##### Unfamiliarity With Information and Communication Technologies

The lack of familiarity with ICT was an obstacle to reach some of the most vulnerable target audience members, and in some cases required a personalized response to help these individuals join and participate. This rang particularly true for projects that addressed the health needs elderly and economically disadvantaged groups:

An older, illiterate woman living with HIV from a very low economic status was very keen in participating [in the virtual support group]…she had difficulties using mobile phones and we had to create a special communication code to ease her use of technology.

##### Data Ownership

Projects that established partnerships with private service providers faced issues with mobile phone user data management and ownership, hampering their ability to establish a fluid communication with target audience members. Deficient mobile network and Internet services in remote areas were also reported:

The messages went into the different mobile providers systems, into their database, and we then were at the mercy of those providers to push that data back to us.

## Discussion

### Principal Results

Among our sample of initiatives, ICT was incorporated as a pivotal element of project implementation for a variety of reasons, most notably the expansion of project geographic coverage, the potential to better access hard-to-reach audiences, perceived low cost, and the improvement of data management and availability. Participant narratives indeed illustrated the edge that ICT can bring to projects addressing the health needs of underserved populations. From improving a country’s ability to respond to a water contamination emergency in an isolated rural village, mobilizing large numbers of people to obtain political support for a largely unattended public health problem, or connecting members of a stigmatized group with much-needed prevention services, participants strongly conveyed the critical role played by ICT in addressing public health issues among these vulnerable groups.

Despite these promising accomplishments, participant narratives equally articulated the significant challenges faced during project implementation.

In an environment where pilot projects seemed to be the norm, sustainability was a clear, forefront concern, particularly when activities were primarily supported by international funding or when teams experienced transitions in leadership or key technical staff. As if tagged with an expiry date, projects often operated within a “pilot mode”, with a strong commitment to achieve clearly defined outputs but without a clearly defined sustainability plan. With the exception of surveillance focused projects, most interventions did not engage actively with local government structures.

The strong drive among projects to engage with a diverse range of mostly local stakeholders—community leaders, academics, government officials, the private sector—can be interpreted as a clear, concerted effort to enhance sustainability through partnerships. It also seems to reveal an acknowledgement of the significant effect that pre-existing, confounding societal factors can exert in project success or failure. Even when eHealth is touted as an innovative departure from traditional public health approaches and relies on novel channels like social media, apps, and text messages, these projects came to operate into pre-existing social systems already shaped by their own nuanced determinants. Participants described how issues like unequal access to ICT among the most vulnerable (often disguised in thriving ICT penetration rates), persistent stigma and discrimination, or demotivated workforces can inherently affect project capacity to make even the smallest dent in reaching their expected health objectives. For instance, several participants described how the most vulnerable often lacked access or familiarity with ICT and reliable Internet or phone services—the very channels for program implementation. In the case of mHealth interventions, the lack of clearly defined data ownership and access impeded their ability to effectively use customer information for project monitoring and communicating back with consenting target audience members.

Although its benefits were widely acknowledged, the perception of social media and mobile phone apps as exclusionary channels lingered among many participants. This was strongly linked to the understanding that use of both of these required access to a computer or smart phone, as well as Internet service—all of which were limited or unavailable to target audiences. This perception, paired with the ubiquitous nature of mobile phones, prompted several projects to choose text messages as the preponderant delivery channel in their interventions. Even in the case of projects whose target audience was composed by tech-savvy youth, who were purportedly more likely to have access to and familiarity with ICT, their motivation behind using these technologies was often unrelated to health; striking their interest and engagement for health-related purposed thus became a challenge. Regarding networking among these projects, geographical proximity seemed to weigh substantially in the awareness of other interventions, even more than other potentially bonding factors like employment of the same ICT tool or working towards tackling a similar public health issue.

### Recommendations

Projects should employ a combination of strategies to overcome the pilot phase where so many currently stumble: addressing sustainability issues from the earliest phases of project design, incorporating program activities into existing government structures, identification of additional sources for renewed funding (including self-generated revenue), and a greater emphasis on capacity building and procedure standardization. mHealth projects establishing partnerships with private service providers should negotiate early on legal issues that could hamper their access to participant data.

Formative research prior to project kick-off and routine monitoring during implementation may help identify potential drawback factors, such as lack of familiarity with ICT or difficulties in establishing meaningful interactions with target audiences. Projects should be ready to design and adapt custom solutions to address these confounding factors.

There seems to be potential for enhanced interaction and collaboration among eHealth practitioners and local stakeholders in close proximity, like policy makers, decision makers, academics, and field enthusiasts. Initiatives promoting public eHealth communities of interest and/or practice could consider a two-tiered approach that stimulates, on the one hand, interaction and potential collaboration with more “local clusters” of eHealth practitioners with a heightened likelihood of some type of face-to-face interaction, while, on the other hand, supporting the integration of these local clusters into a broader, regional network. The likelihood of local clusters emerging and operating regularly would likely be influenced by the context of eHealth support and practice in each city or country.

### Limitations

This research paper is based on in-depth interviews with eight projects and results cannot be extrapolated to all interventions of this nature in LAC. Results are based on participant narratives that may include biases of memory selectiveness and attribution. While all organizations that implemented the selected projects were still operating at the time of the interview, two of the projects were completed more than 2 years ago, so there is a possible memory bias in some interviews. Most interviews were conducted in Spanish. Thus, a translation bias may have occurred when citing quotes, which have been translated to English.

Areas for future research include a deeper analysis of eHealth´s potential to reduce health inequities within protractedly unequal societies, the operationalization of equity in ICT-supported health projects, and the exploration of alternative models of sustainability for projects of this nature, including the feasibility of self-generated revenue.

### Comparison With Prior Work

The potential and benefits of the use of ICT in health have been extensively touted and helped support the unbridled optimism that has often surrounded the early stages of design and implementation of eHealth initiatives in developing countries [2,6]. Mirroring results from our study, ICT have been credited with increasing reach and raising awareness of health issues among hard to reach groups and offering a more cost-effective way to provide tailored services [23]. From a LAC perspective, most publications have focused on the experience of a specific intervention, particularly in the telehealth field, rather than on a collective perspective on common challenges and perceived impact on equity, which we believe is the main contribution of this paper. While positive short-term outputs and outcomes of eHealth interventions in LAC have been reported in several studies, the analysis of project impact on health inequities and sustainability are still mostly unexplored and additional research on these topics has been strongly advocated [24,25]. The lack of exposure to computer/Internet technology among vulnerable groups and the need for broader partnerships to guarantee project survival beyond pilot phases have also been evidenced earlier and are aspects that need to be addressed in equity-focused health interventions [19,26-28].

### Conclusions

ICT may contribute to improved outcomes for projects addressing the health needs of vulnerable populations by expanding geographic coverage, increasing reach into marginalized or hard-to-reach groups, allowing real-time data collection and transforming target audiences from passive recipients into content disseminators and creators. While most projects did not include the concept of health equity as an explicit project component, they clearly perceived their contributions to health equity in the provision of health information and linkage to health services among members of groups suffering from greater morbidity because of poverty, remote place of residence, lack of relevant public programs, and/or stigma and discrimination, and in more timely responses by authorities to the health needs of these groups as a result of the increased availability of strategic information on morbidity and its social determinants. Projects tended to be small pilot interventions with limited engagement with the formal health sector. Their financial and technical sustainability was threatened by reliance on external funding and weak transitional structures within the organizations. Collaborative projects with government institutions seemed most optimistic about sustainability. Projects experienced challenges in establishing meaningful communication with target audience members, mainly because of divergent motivations behind ICT use between projects and its target audience and the lack of access or familiarity with ICT among the most underserved members of such audiences.

## Abbreviations

eSAC: Public eHealth, Innovation & Equity in Latin America and the Caribbean project
ICT: Information and communication technologies
LAC: Latin America and the Caribbean
WHO: World Health Organization

## References

[1] Brennan Ramirez LK, Baker EA, Metzler M (2008). Promoting Health Equity: A Resource to Help Communities Address Social Determinants of Health.

[2] Braveman P, Gruskin S (2003). Defining equity in health. J Epidemiol Community Health.

[3] Regional human development report for Latin America and the Caribbean 2010 (2010). Acting on the future: breaking the intergenerational transmission of inequality.

[4] Gasparini L, Lustig N (2011). The rise and fall of income inequality in Latin America in Ocampo J, Ros J. editors. Oxford Handbook of Latin American Economics.

[5] Latin American Center for Rural Development (2012). Poverty and inequality Latin American Report 2011.

[6] Blaya Joaquin A, Fraser Hamish S F, Holt Brian (2010). E-health technologies show promise in developing countries. Health Aff (Millwood).

[7] Gerber Ticia, Olazabal Veronica, Brown Karl, Pablos-Mendez Ariel (2010). An agenda for action on global e-health. Health Aff (Millwood).

[8] Sinha C, Garro-Strauss D (2013). Research on eHealth across Health Systems: Contributions to Strengthen a Field in Elder, L, Emdon, H, Fuchs, R, Petrazzini, B. Connecting ICTs to Development: The IDRC Experience.

[9] Fair Society, Healthy Lives (2010). Strategic Review of Health Inequities in England.

[10] Closing the gap in a generation: health equity through action on the social determinants of health (2008). Geneva, Switzerland.

[11] Blas E, Sivasankara-Kurup A (2010). Equity, social determinants and public health programmes.

[12] Friede A, Blum H L, McDonald M (1995). Public health informatics: how information-age technology can strengthen public health. Annu Rev Public Health.

[13] Hoffman Jeffrey A, Cunningham Janice R, Suleh Andrew J, Sundsmo Aaron, Dekker Debra, Vago Fred, Munly Kelly, Igonya Emmy Kageha, Hunt-Glassman Jonathan (2010). Mobile direct observation treatment for tuberculosis patients: a technical feasibility pilot using mobile phones in Nairobi, Kenya. Am J Prev Med.

[14] Chang Larry W, Kagaayi Joseph, Nakigozi Gertrude, Packer Arnold H, Serwadda David, Quinn Thomas C, Gray Ronald H, Bollinger Robert C, Reynolds Steven J (2008). Responding to the human resource crisis: peer health workers, mobile phones, and HIV care in Rakai, Uganda. AIDS Patient Care STDS.

[15] Mahmud Nadim, Rodriguez Joce, Nesbit Josh (2010). A text message-based intervention to bridge the healthcare communication gap in the rural developing world. Technol Health Care.

[16] Aranda C, Mohutsiwa-Dibe N, Loukanova S (2014). Systematic review on what works, what does not work and why of implementation of mobile health (mHealth) projects in Africa. BMC Public Health.

[17] Rice RE, Rice R, Katz J (2001). The Internet and health communication: a framework of experiences. The Internet and Health Communication.

[18] Lewis Trevor, Synowiec Christina, Lagomarsino Gina, Schweitzer Julian (2012). E-health in low- and middle-income countries: findings from the Center for Health Market Innovations. Bull World Health Organ.

[19] Gurak L, Hudson B, Murero M, Rice R (2006). E-health: Beyond internet searches. The Internet and Health Care: Theory, Research, and Practice.

[20] Fernandez A, Oviedo E (2010). ICT in the health sector. Social policy series Number 165.

[21] Bellagio eHealth Evaluation Group (2011). Consensus statement of the WHO Global eHealth Evaluation Meeting, Bellagio.

[22] Knol Anne B, Slottje Pauline, van der Sluijs Jeroen, Lebret Erik (2010). The use of expert elicitation in environmental health impact assessment: a seven step procedure. Environ Health.

[23] Minichiello Victor, Rahman Saifur, Dune Tinashe, Scott John, Dowsett Gary (2013). E-health: potential benefits and challenges in providing and accessing sexual health services. BMC Public Health.

[24] Glasgow Russell E, Phillips Siobhan M, Sanchez Michael A (2014). Implementation science approaches for integrating eHealth research into practice and policy. Int J Med Inform.

[25] Schnall Rebecca, Travers Jasmine, Rojas Marlene, Carballo-Diéguez Alex (2014). eHealth interventions for HIV prevention in high-risk men who have sex with men: a systematic review. J Med Internet Res.

[26] Choi Namkee G, Dinitto Diana M (2013). The digital divide among low-income homebound older adults: Internet use patterns, eHealth literacy, and attitudes toward computer/Internet use. J Med Internet Res.

[27] Tomlinson M, Rotheram-Borus MJ, Swartz L (2013). Tsai AC: Scaling Up mHealth: Where Is the Evidence?. PLoS Med.

[28] Lemaire J (2010). Scaling up mobile health: Elements necessary for the successful scale up of mHealth in developing countries.

